# Cyber-ostracism and academic engagement among Chinese university students: a three-wave longitudinal study of mediating and moderating mechanisms

**DOI:** 10.3389/fpsyg.2026.1867464

**Published:** 2026-09-23

**Authors:** Jie Wu, BenYu Guo

**Affiliations:** 1 School of Education, Jiangsu Open University, Nanjing, Jiangsu, China; 2 School of Psychology, Nanjing Normal University, Nanjing, Jiangsu, China

**Keywords:** academic engagement, cyber-ostracism, psychological resilience, self-esteem, social avoidance, social support

## Abstract

Cyber-ostracism has become an important challenge in digitally mediated university life, yet its influence on academic engagement remains insufficiently understood. This study examined the longitudinal relationship between cyber-ostracism and academic engagement among Chinese university students, as well as the mediating roles of self-esteem and social avoidance and the moderating roles of social support and psychological resilience. A three-wave survey was conducted with 450 undergraduates from a provincial public university in China. Structural equation modeling showed that cyber-ostracism at Time 1 significantly predicted lower academic engagement at Time 3 (*β* = −0.28, *p* < 0.001). Indirect effects through self-esteem (β = −0.16, 95% CI [−0.23, −0.10]) and social avoidance (*β* = −0.12, 95% CI [−0.19, −0.06]) were significant. A further indirect effect through self-esteem and social avoidance was also significant (*β* = −0.08, 95% CI [−0.13, −0.04]), consistent with the hypothesized ordering from lower self-esteem to greater social avoidance, although temporal precedence between the two T2 mediators cannot be established. Social support (*β* = 0.12, *p* = 0.003) and psychological resilience (*β* = 0.10, *p* = 0.012) significantly buffered the negative effect of cyber-ostracism. The findings indicate that online exclusion may undermine students’ learning involvement through both direct and indirect pathways. Strengthening supportive learning climates and students’ coping resources may help sustain engagement in digital higher education contexts.

## 1 Introduction

In recent years, online interactions have become an essential part of university students’ academic and social lives. While digital platforms facilitate communication and learning, they also expose students to negative interpersonal experiences such as cyber-ostracism. Cyber-ostracism refers to being ignored, excluded, or rejected in online contexts, such as receiving no responses in group chats, having social media posts ignored, or being removed from online communities (Williams et al., 2000; Tan, 2025). Although extensive research has examined face-to-face ostracism, cyber-ostracism remains underexplored despite its prevalence and potential psychological consequences (Tan, 2025; Xing and Kuo, 2024). Existing studies have mainly focused on immediate outcomes such as emotional distress, anxiety, and aggression (Williams et al., 2000; Hao, 2025), while its long-term impact on academic engagement has received limited attention.

The effects of cyber-ostracism can be understood through the Need Threat Model, which proposes that ostracism threatens four fundamental psychological needs: belonging, self-esteem, control, and meaningful existence (Williams, 2001). These need-threatening effects can also occur in computer-mediated contexts. Experimental evidence indicates that even brief experiences of ostracism in virtual interactions can reduce individuals’ perceived belonging, self-esteem, control, and meaningful existence (Zadro et al., 2004). Cyber-ostracism may be particularly ambiguous because online interactions are characterized by asynchronicity, dependence on technology, and a lack of social cues, making it difficult for individuals to determine whether and why they are being ignored or excluded (Niu et al., 2018). Despite this ambiguity, cyber-ostracism remains psychologically distressing and is associated with adverse emotional outcomes. For example, Niu et al. (2018) found that cyber-ostracism was positively associated with depression among Chinese adolescents, with optimism buffering this adverse association.

Academic engagement broadly refers to students’ active and sustained involvement in learning and has been conceptualized from multiple perspectives. For example, Fredricks et al. (2004) described engagement in terms of behavioral, emotional, and cognitive dimensions, providing a broad framework for understanding students’ participation in academic activities. In the present study, however, academic engagement is conceptualized more specifically in accordance with the UWES-S framework as a positive, fulfilling study-related state characterized by vigor, dedication, and absorption (Schaufeli et al., 2002). These dimensions reflect students’ energy and persistence, sense of commitment and significance, and concentrated involvement in academic activities, respectively. Although this conceptualization is related to the broader multidimensional engagement framework, the two approaches are not treated as interchangeable. Higher academic engagement is generally associated with favorable academic and psychological outcomes (Fredricks et al., 2004; Wu et al., 2023). Moreover, perceived social support and a sense of belonging are positively associated with student engagement, suggesting that experiences of social exclusion may be longitudinally associated with lower motivation and involvement in academic activities (Totura et al., 2014).

Despite these theoretical links, longitudinal evidence on cyber-ostracism and academic engagement among university students remains limited. Prior adolescent research indicates that peer exclusion negatively affects engagement through psychological distress and reduced motivation (Totura et al., 2014). Similarly, cyber-ostracism may reduce university students’ engagement via lowered self-esteem and increased social avoidance. A longitudinal design is therefore necessary to capture dynamic and potential causal relationships.

Accordingly, this study examines the longitudinal association between cyber-ostracism and academic engagement among Chinese university students. It specifically investigates (1) whether cyber-ostracism prospectively predicts changes in academic engagement, (2) whether self-esteem and social avoidance represent psychological pathways linking cyber-ostracism with subsequent engagement, and (3) whether social support and psychological resilience moderate these associations. The study seeks to extend the Need Threat Model beyond a simple contextual application by considering how the distinctive affordances of digital communication may shape experiences of ostracism. In contrast to many face-to-face interactions, online exclusion frequently occurs through asynchronous, cue-reduced, and ambiguous signals, such as delayed responses, unanswered messages, or exclusion from digital exchanges. From a cues-filtered-out perspective, the reduced availability of nonverbal and contextual information in computer-mediated communication may increase uncertainty about the meaning and intentionality of exclusion, while the persistent accessibility of digital communication may allow exclusion-related experiences to extend beyond a single interaction. Thus, digital environments are conceptualized not merely as another setting in which ostracism occurs, but as a communication context that may shape how exclusion is interpreted and how need threat is sustained. By further distinguishing self-esteem as an intrapersonal self-evaluative pathway and social avoidance as an interpersonal withdrawal pathway, the study provides a more differentiated account of how digitally experienced need threat is longitudinally associated with academic engagement.

## 2 Literature review

### 2.1 Conceptualizing cyber-ostracism in digital learning contexts

Cyber-ostracism refers to the experience of being ignored, excluded, or left unanswered in online interactions. In university settings, it may take seemingly ordinary but psychologically meaningful forms, such as receiving no response to a message in a class group, having one’s opinion overlooked in an online discussion, or being excluded from a shared digital space. Ostracism research suggests that such experiences can function as signals of relational devaluation and are closely associated with threats to individuals’ fundamental social needs, particularly the need to belong (Williams et al., 2000; Williams, 2007). As students increasingly rely on messaging applications, social platforms, and learning management systems, cyber-ostracism represents a relevant interpersonal stressor in digitally mediated learning environments.

The primary theoretical framework for understanding these experiences is the Need Threat Model, which proposes that ostracism can threaten four fundamental needs: belonging, self-esteem, control, and meaningful existence (Williams, 2001; Williams, 2007). Threats to these needs are associated with psychological distress and a range of responses aimed at managing or restoring threatened social needs, including heightened vigilance, withdrawal, and compensatory behavior. Research extending this framework to digital contexts similarly suggests that online exclusion is associated with emotional and behavioral responses. For example, cyber-ostracism has been associated with aggressive behavior among university students, with basic psychological need satisfaction identified as a potential mediating process and self-integrity as a protective factor (Xing and Kuo, 2024). Likewise, relatively subtle online exclusion cues, such as receiving “dislikes” or being ignored, have been linked to need threat and subsequent coping responses (Lutz and Schneider, 2021).

Digital communication may nevertheless shape how ostracism is experienced and interpreted. From a cues-filtered-out perspective, computer-mediated interactions provide fewer nonverbal and contextual cues than face-to-face communication, making the intentions underlying social behavior more difficult to interpret. In digital learning environments, unanswered messages, delayed responses, or exclusion from online exchanges may therefore create uncertainty about whether exclusion is intentional. Moreover, because interactions can continue across class groups, learning platforms, and social networks beyond a single physical encounter, exclusion-related cues may remain psychologically salient over time. These characteristics suggest that cyber-ostracism should not be viewed simply as offline ostracism transferred to an online setting; rather, the affordances of digital communication may shape the interpretation and persistence of need-threatening experiences.

Although the Need Threat Model encompasses all four fundamental needs, the present study focuses specifically on self-esteem as one theoretically relevant self-evaluative pathway. Cyber-ostracism may convey negative information about an individual’s perceived social value and acceptance, making self-esteem particularly relevant to understanding subsequent academic involvement. Importantly, this selective focus does not imply that self-esteem is more central than the other threatened needs. Belonging is especially relevant to ostracism but was not directly measured in the present study. Accordingly, self-esteem is examined as one component of the broader need-threat response rather than as an exhaustive representation of the Need Threat Model.

A complementary explanation can be derived from Conservation of Resources (COR) theory, which proposes that individuals strive to acquire, maintain, and protect valued psychological and social resources and that initial resource loss may increase vulnerability to subsequent losses (Hobfoll, 1989, 2001). In the context of cyber-ostracism, threats to self-worth and social connection may diminish psychological resources available for sustained academic effort, while social withdrawal may further restrict opportunities to obtain compensatory resources through peer interaction. From this perspective, self-esteem can be understood as an important personal resource, whereas social interaction provides access to interpersonal resources that may support coping and academic functioning. COR theory therefore complements the Need Threat Model by helping explain why experiences of digital exclusion may be longitudinally associated with subsequent academic engagement.

### 2.2 Psychological consequences of cyber-ostracism

A growing body of research indicates that cyber-ostracism has adverse psychological consequences. Across studies, excluded individuals report lower well-being, greater distress, and increased emotional reactivity. In a moderated mediation study of college students, cyber-ostracism was associated with poorer well-being through lower need satisfaction and higher psychological stress, suggesting that online exclusion is not merely a fleeting interpersonal episode but a meaningful stressor with broader mental health implications (Wang et al., 2022). In another recent study, cyber-ostracism predicted aggressive behavior among undergraduates, again highlighting that online exclusion can trigger maladaptive emotional and behavioral responses rather than simple temporary discomfort (Xing and Kuo, 2024).

Self-esteem appears to be one of the most important psychological resources affected by exclusion. According to the Need Threat Model, ostracism communicates relational devaluation, which can reduce perceived self-worth and weaken confidence in one’s social value. Recent Chinese evidence supports this pathway. Lei et al. (2024) found that ostracism was positively associated with social withdrawal among Chinese adolescents, and that self-esteem partially mediated this association. Although that study focused on adolescents rather than university students, it provides important support for the idea that exclusion can erode self-evaluation and then promote withdrawal. Earlier Chinese research also showed that both real-life and online social exclusion were associated with depressive symptoms among university students, with self-control functioning as a mediating mechanism (Sun et al., 2017). Together, these findings suggest that cyber-ostracism may affect students not only emotionally, but also through self-related and self-regulatory processes that influence later behavior.

Social withdrawal and avoidance are another well-established consequence of exclusion. Ostracized individuals often reduce interpersonal contact to protect themselves from further rejection, but this coping pattern can backfire by further limiting support and participation. Recent work on Chinese adolescents found that ostracism directly predicted social withdrawal and indirectly did so through self-esteem threat (Lei et al., 2024). In the university context, social withdrawal may be especially harmful because academic success increasingly depends on collaboration, discussion, and help-seeking, especially in digitally mediated learning environments. When students retreat from peers after exclusion, they may also withdraw from academic interaction, thereby reducing access to information, feedback, and emotional support.

### 2.3 Academic engagement as a key educational outcome

Academic engagement has been conceptualized from multiple theoretical perspectives. One influential framework proposed by Fredricks et al. (2004) distinguishes behavioral, emotional, and cognitive engagement, referring, respectively, to students’ participation and persistence, affective involvement, and cognitive investment in learning. In the present study, however, academic engagement is operationalized within the UWES-S framework, which conceptualizes engagement as a positive and fulfilling study-related state comprising vigor, dedication, and absorption (Schaufeli et al., 2002). Although the two perspectives share an emphasis on students’ active involvement in learning, they differ in conceptual focus and are not treated as interchangeable. Accordingly, the UWES-S framework provides the primary theoretical and operational basis for assessing academic engagement in this study.

Academic engagement represents an important educational outcome because it is closely associated with students’ academic achievement, persistence, and well-being (Fredricks et al., 2004). Rather than being determined solely by individual motivation, engagement is embedded within students’ broader social and psychological environments. Supportive interpersonal relationships can provide emotional and motivational resources that facilitate sustained involvement in learning, whereas rejection, disconnection, and insufficient social support may undermine students’ willingness and capacity to remain actively engaged. This social-contextual perspective is particularly relevant to the present study because cyber-ostracism represents a form of digital social exclusion that may disrupt the psychological resources needed to sustain study-related engagement.

Empirical evidence increasingly supports the importance of such social and psychological resources for academic engagement, including among Chinese students. For example, Chen et al. (2023) reported a positive association between social support and academic engagement, with life satisfaction and academic motivation accounting for significant indirect relationships. Similarly, Wang and Wang (2025) found that self-compassion and social support were positively associated with academic engagement and that stress management partially accounted for these relationships. Together, these findings suggest that students’ engagement is related not only to supportive external environments but also to the psychological resources through which they respond to academic and interpersonal demands.

Among these internal resources, academic self-efficacy and psychological resilience appear particularly relevant. Wang and Zhang (2024) reported positive relationships between academic self-efficacy, psychological resilience, professional commitment, and learning engagement, highlighting the role of personal psychological strengths in maintaining academic involvement. Research among younger Chinese students has similarly identified a positive association between psychological resilience and learning engagement across dimensions corresponding to vigor, dedication, and absorption. Although these younger populations differ developmentally from university students, such findings provide complementary evidence that resilience may help individuals maintain active involvement in learning when confronted with difficulties or adverse experiences.

Taken together, this literature suggests that academic engagement is shaped by the interplay between students’ social experiences and their internal psychological resources. This perspective is especially pertinent in contemporary higher education, where students increasingly navigate academic and interpersonal interactions through digital environments while simultaneously facing substantial demands for autonomy and self-regulation. Cyber-ostracism may therefore be prospectively associated with lower academic engagement by disrupting students’ social and psychological resources, whereas resilience may represent a personal resource relevant to maintaining engagement under such adverse conditions. Within this framework, academic engagement constitutes a theoretically meaningful educational outcome through which the longer-term academic implications of cyber-ostracism can be examined.

### 2.4 Linking cyber-ostracism to academic engagement

On theoretical grounds, cyber-ostracism should negatively predict academic engagement. If online exclusion threatens belonging and self-worth, students may become less motivated to participate in academic tasks, less willing to contribute to group learning, and less emotionally invested in the learning process. The Need Threat Model explains this by suggesting that exclusion initially activates distress and social monitoring, which can shift cognitive resources away from learning and toward self-protection (Williams, 2007; Baumeister et al., 2005). In a digital university environment, where peer interaction often occurs through group chats, forums, and virtual class platforms, the experience of being ignored may also reduce students’ sense that they have a meaningful place in the academic community.

Recent evidence supports this expectation indirectly. Studies of Chinese university students have shown that cyber-ostracism is linked to poorer psychological adjustment through need frustration and stress (Wang et al., 2022), and to more aggressive behavioral responses through basic psychological needs and self-integrity (Xing and Kuo, 2024). These findings imply that cyber-ostracism is capable of altering not only emotion, but also students’ self-regulation and interpersonal orientation. Because academic engagement depends on sustained attention, willingness to cooperate, and continued participation, these exclusion-related processes are likely to undermine engagement over time.

Chinese studies on related forms of social exclusion provide further support. For example, Jin et al. (2019) found that network social exclusion predicted both online aggression and traditional aggression among university students, with alienation acting as a mediator. Although aggression is not the same as disengagement, the study demonstrates that online exclusion can meaningfully shape the functioning of Chinese college students. Taken together with the evidence linking social support to engagement and resilience to learning outcomes, it is reasonable to infer that cyber-ostracism may have an equally important but previously underexamined effect on academic engagement.

### 2.5 Mediating mechanisms: self-esteem and social avoidance

Self-esteem is a central mediator in the cyber-ostracism–engagement link. Ostracism reduces perceived social value, and lower self-esteem can reduce students’ confidence to participate, ask questions, or persist with difficult tasks. This mechanism is consistent with a large body of ostracism research showing that exclusion threatens self-evaluation and self-regulation (Baumeister et al., 2005; Williams, 2007). Chinese evidence further supports this pathway: Lei et al. (2024) found that ostracism increased social withdrawal partly by lowering self-esteem. The implication for academic engagement is clear: once self-esteem is damaged, students may interpret academic challenges more negatively and become less willing to invest effort in learning activities.

Social avoidance is the second important mediator. After exclusion, students may reduce social contact to avoid further hurt, but this protective strategy can have academic costs because it limits participation in discussions, peer learning, and collaborative problem solving. Recent ostracism studies in China show that exclusion predicts social withdrawal directly and through self-esteem threat, reinforcing the plausibility of avoidance as a behavioral pathway (Lei et al., 2024). In higher education, social avoidance may be particularly consequential because many learning tasks require coordination, discussion, and mutual feedback. Thus, cyber-ostracism may lower engagement not only by reducing internal confidence, but also by reshaping students’ interaction patterns in ways that isolate them from the academic community.

### 2.6 Moderating roles of social support and psychological resilience

Although cyber-ostracism may be harmful, its effects are unlikely to be uniform across all students. Social support is one of the most established buffering resources in the literature. When students feel emotionally supported by family, friends, teachers, or peers, the sting of exclusion is less likely to undermine their academic functioning. Consistent with the buffering hypothesis, support can restore belonging, reduce stress appraisal, and help students maintain motivation despite negative social experiences (Cohen and Wills, 1985). Recent empirical work supports this idea in higher education: Chinese university studies have found that social support is positively associated with academic engagement and that it operates both directly and indirectly through life satisfaction, academic motivation, and stress management (Chen et al., 2023; Wang and Wang, 2025).

Psychological resilience is a pivotal protective factor in student academic functioning. It broadly refers to individuals’ ability to adapt positively to adversity and recover adaptive functioning following stress. In higher education contexts, resilience facilitates emotion regulation, sustained academic effort, and goal persistence under pressure. Recent empirical studies among Chinese university students support its academic benefits. Specifically, Cai and Meng (2025) found that academic resilience positively predicts academic performance, with teacher support mediating this relationship. Consistently, Wang and Zhang (2024) reported a positive association between psychological resilience and learning engagement, demonstrating that resilience operates interactively with academic self-efficacy and professional commitment. Collectively, these findings indicate that resilience is not merely a dispositional trait but a functional psychological resource that helps students maintain learning engagement amid academic stress and uncertainty.

In combination, social support and psychological resilience suggest a resource-based account of cyber-ostracism. Cyber-ostracism may drain social and psychological resources, but students who possess strong support networks and higher resilience may be better able to preserve engagement. This perspective is reinforced by a recent Chinese study on university students in which self-compassion and social support were positively linked to academic engagement, and stress management partly explained the relationship (Wang and Wang, 2025). It is also consistent with research showing that online exclusion and digital stress can affect students differently depending on the availability of supportive relationships and adaptive coping capacities.

### 2.7 Research gaps

Despite this growing literature, several gaps remain. First, most cyber-ostracism studies have focused on short-term emotional or behavioral outcomes, such as aggression, stress, depression, or withdrawal, whereas the longitudinal effect on academic engagement has received far less attention. Second, prior work often examines only one psychological pathway at a time. There is still limited integrated evidence showing how cyber-ostracism may reduce academic engagement through both self-esteem and social avoidance. Third, although recent Chinese studies have highlighted the importance of social support and resilience for engagement, these resources have rarely been tested together in a single model of cyber-ostracism. Finally, much of the available evidence comes from adolescents or mixed-age samples, while university students occupy a distinct developmental and educational stage with more autonomy, more online learning demands, and potentially greater sensitivity to digital peer exclusion. These gaps justify the present study’s focus on the longitudinal pathways through which cyber-ostracism may shape academic engagement among Chinese university students.

## 3 Conceptual framework and research hypotheses

### 3.1 Conceptual framework

Based on the literature reviewed, the present study proposes a conceptual framework that examines the longitudinal relationship between cyber-ostracism and academic engagement among university students. The framework integrates theoretical perspectives from the Need Threat Model (Williams, 2001) and academic engagement theory (Fredricks et al., 2004; Yang, 2024). According to the Need Threat Model, social exclusion—whether offline or online—threatens individuals’ basic psychological needs for belonging, self-esteem, control, and meaningful existence. Threats to these needs can trigger negative emotional and behavioral responses, including social withdrawal, avoidance behaviors, and reduced motivation (Williams and Zadro, 2005; Shi et al., 2025).

Academic engagement is conceptualized as a positive and fulfilling psychological state related to students’ academic activities. Based on the Utrecht Work Engagement Scale–Student version (UWES-S) (Schaufeli et al., 2002; Yang, 2024), it comprises three dimensions: vigor, dedication, and absorption. Vigor reflects energy and persistence in academic work; dedication refers to enthusiasm, significance, and commitment to one’s studies; and absorption describes deep concentration and immersion in academic activities. Prior research indicates that negative social experiences, such as peer exclusion or cyber-ostracism, can reduce engagement through both direct and indirect pathways (Niu et al., 2018; Totura et al., 2014).

The conceptual framework proposes that cyber-ostracism (T1) affects academic engagement (T3) both directly and indirectly through psychological mediators such as self-esteem (T2) and social avoidance (T2). Additionally, the framework posits that contextual moderators, including social support and psychological resilience, can buffer the negative effects of cyber-ostracism on engagement (Wu et al., 2023; Shi et al., 2022).

### 3.2 Research hypotheses

Based on the conceptual framework and existing empirical evidence, the following hypotheses are proposed:

*H1*: Cyber-ostracism at Time 1 negatively predicts academic engagement at Time 3, controlling for baseline engagement.

Rationale: Previous studies indicate that exclusion experiences reduce students’ motivation and participation in learning, suggesting a direct negative relationship between cyber-ostracism and engagement (Williams et al., 2000; Tan, 2025).

*H2*: Self-esteem mediates the relationship between cyber-ostracism and academic engagement.

Rationale: Cyber-ostracism threatens individuals’ self-worth, which in turn undermines motivation and engagement in academic tasks (Niu et al., 2018; Williams, 2001).

*H3*: Social avoidance mediates the relationship between cyber-ostracism and academic engagement.

Rationale: Students experiencing cyber-ostracism are likely to withdraw from social interactions, reducing opportunities for academic collaboration and engagement (Shi et al., 2025; Ding et al., 2025).

*H4*: Social support moderates the effect of cyber-ostracism on academic engagement, such that high levels of social support weaken the negative effect.

Rationale: Supportive social networks provide emotional resources that can buffer the psychological threats posed by cyber-ostracism (Wu et al., 2023; Tan, 2025).

*H5*: Psychological resilience moderates the effect of cyber-ostracism on academic engagement, such that students with higher resilience experience smaller declines in engagement.

Rationale: Resilient students are better able to cope with social threats and maintain learning motivation despite negative online experiences (Shi et al., 2022; Shi et al., 2025).

*H6*: Cyber-ostracism at T1 is indirectly associated with academic engagement at T3 through a hypothesized sequential pathway involving lower self-esteem and greater social avoidance at T2.

Rationale: Cyber-ostracism may simultaneously lower self-esteem and increase social avoidance, which together reduce students’ emotional, cognitive, and behavioral engagement (Niu et al., 2018; Totura et al., 2014).

## 4 Methodology

### 4.1 Research design

This study employed a three-wave longitudinal survey design to examine the prospective association between cyber-ostracism and academic engagement over time. Data were collected at three measurement occasions (T1, T2, and T3), with an interval of approximately 5 months between successive waves. This interval was selected to approximate the duration of an academic semester, allowing sufficient time for meaningful variation in students’ online social experiences, self-evaluative responses, social withdrawal tendencies, and academic engagement to emerge while maintaining continuity within the university context. The interval also helped reduce the likelihood that the observed associations primarily reflected short-term reactions to isolated online interactions. The temporal separation of the predictor, mediators, and outcome was therefore intended to strengthen temporal ordering and reduce potential bias associated with single-wave measurement.

The primary analytical framework was a three-wave longitudinal path model specified according to the theoretically hypothesized temporal sequence. Cyber-ostracism at T1 served as the primary predictor, self-esteem and social avoidance at T2 were examined as mediating mechanisms, and academic engagement at T3 served as the primary outcome. Baseline levels of the relevant endogenous constructs were controlled to account for temporal stability. Rather than testing fully reciprocal cross-lagged relationships, the model focused on theoretically specified prospective associations. Because the study was observational rather than experimental, the longitudinal design strengthens temporal inference but does not establish definitive causal relationships.

### 4.2 Participants and data collection procedures

Participants were undergraduate students recruited from a provincial public university in China. Approximately 600 students were invited to participate at baseline, of whom 520 provided usable questionnaires at Time 1 (T1). The same cohort was followed at two subsequent measurement occasions, with anonymous identification codes used to match responses across waves. Of the 520 valid T1 participants, 487 participated at T2 and 458 at T3.

Before analysis, questionnaires were screened using prespecified data-quality criteria. Responses were excluded if they contained more than 20% missing data, showed clear invariant or patterned responding, had implausibly short completion times, or could not be reliably matched across waves. Following final screening and longitudinal matching, eight additional cases were excluded, resulting in a final analytic sample of 450 participants with valid matched data across all three waves. This represented a retention rate of 86.5% relative to the valid T1 sample. Attrition primarily reflected nonparticipation in follow-up assessments, withdrawal, or incomplete questionnaires; where no specific reason was documented, cases were classified as loss to follow-up for an unknown reason.

An *a priori* power analysis was conducted for the moderation analyses. Assuming a small interaction effect (*f^2^* = 0.02), *α* = 0.05, and power = 0.80, the estimated minimum sample size was approximately 395 participants (Cohen, 1988; Faul et al., 2009). Thus, the final sample of 450 exceeded the required threshold and provided adequate statistical power for the hypothesized interaction effects.

### 4.3 Measures

All measures were administered at the relevant measurement occasions according to the three-wave longitudinal design. To ensure consistency between the measurement descriptions and the variables used in the statistical analyses, the response coding and composite-score calculation procedures are explicitly reported below. Unless otherwise specified, composite scores were calculated by averaging the corresponding item scores, with higher scores representing higher levels of the respective constructs. The same scoring and coding procedures were used for the descriptive statistics, reliability analyses, correlation analyses, and subsequent longitudinal structural analyses.

#### 4.3.1 Cyber-ostracism

Cyber-ostracism was assessed using the Cyber-Ostracism Questionnaire (COQ) developed by Niu et al. (2018). The questionnaire consists of 14 items covering three dimensions: one-on-one online exclusion, group chat exclusion, and personal online space exclusion. Items were rated on a 5-point Likert-type scale ranging from 1 (never) to 5 (always). Item scores were averaged to obtain dimension and overall scores, with higher scores indicating more frequent experiences of cyber-ostracism. Previous research has demonstrated satisfactory reliability of the COQ in Chinese samples (Cronbach’s α > 0.80; Niu et al., 2018).

#### 4.3.2 Academic engagement

Academic engagement was assessed using the Utrecht Work Engagement Scale–Student version (UWES-S) (Schaufeli et al., 2002; Yang, 2024), which assesses vigor, dedication, and absorption across 17 items. Although the original UWES-S uses seven response categories coded from 0 (never) to 6 (always), the response categories in the present study were coded from 1 (never) to 7 (always), while retaining the same seven-category ordering. Item scores were averaged to obtain an overall academic engagement score, with higher scores indicating greater academic engagement. This 1–7 coding scheme was used consistently in the descriptive and subsequent statistical analyses. Previous research has demonstrated high internal consistency of the UWES-S among Chinese university students (α ≈ 0.88; Meng and Jin, 2017).

#### 4.3.3 Mediators

Self-esteem. Self-esteem was assessed using the 10-item Rosenberg Self-Esteem Scale (RSES; Rosenberg, 1965), which measures global self-worth. Items were scored on a 4-point response scale ranging from 0 (strongly disagree) to 3 (strongly agree). Negatively worded items were reverse-coded where appropriate, after which the item scores were averaged to obtain an overall self-esteem score. Accordingly, possible composite scores ranged from 0 to 3, with higher scores indicating higher levels of self-esteem. Previous studies have demonstrated satisfactory reliability of the RSES in Chinese samples (Cronbach’s α > 0.80; Tian, 2006).

Social avoidance. Social avoidance was assessed using a 14-item measure adapted from the Social Avoidance and Distress Scale (SADS) (Watson and Friend, 1969), with reference to the recent adaptation reported by Zhu and Zhao (2026). The original SADS provides the conceptual and item basis for assessing individuals’ tendencies to avoid social interactions and experience discomfort in interpersonal situations, whereas Zhu and Zhao’s (2026) adaptation informed the use of a 5-point Likert response format. Accordingly, participants rated each item on a scale ranging from 1 (strongly disagree) to 5 (strongly agree), rather than using the dichotomous true/false format of the original SADS. Zhu and Zhao (2026) reported adequate internal consistency for their adapted measure in a pilot sample (Cronbach’s α = 0.84). In the present study, item scores were averaged after reverse-scoring where appropriate, with higher scores indicating greater social avoidance.

#### 4.3.4 Moderators

Social support. Perceived social support was assessed using the Multidimensional Scale of Perceived Social Support (MSPSS) (Zimet et al., 1988), which measures perceived support from family, friends, and significant others. The 12 items were rated on a 7-point Likert scale ranging from 1 (very strongly disagree) to 7 (very strongly agree). Item scores were averaged to obtain an overall perceived social support score, with higher scores indicating greater perceived availability of social support. The Chinese version has demonstrated excellent internal consistency among university students (α > 0.90; Yang et al., 2024).

Psychological resilience. Psychological resilience was assessed using the 25-item Connor–Davidson Resilience Scale (CD-RISC) (Connor and Davidson, 2003). The original CD-RISC employs five response categories coded from 0 to 4; in the present study, these categories were coded from 1 to 5 while preserving their original ordering. Item scores were averaged to calculate the overall psychological resilience score, with higher scores indicating greater capacity to adapt to and recover from adversity. This 1–5 coding scheme was applied consistently throughout the statistical analyses. The Chinese adaptation of the CD-RISC has demonstrated satisfactory reliability and validity in adolescent and young adult samples (α > 0.85; Yu et al., 2011).

#### 4.3.5 Control variables

Demographic and background variables included age, gender, academic year, major, family socioeconomic status, and average daily internet use. These variables were included as covariates to account for potential demographic and contextual confounding effects (Tan, 2025).

### 4.4 Data analysis plan

The data were analyzed using SPSS and Mplus. Preliminary analyses included descriptive statistics, Pearson correlations, and reliability estimates using Cronbach’s alpha and McDonald’s omega. Potential attrition bias was examined by comparing participants retained across all three waves with valid T1 participants who were not retained, using independent-samples t tests for continuous baseline variables and chi-square tests for categorical characteristics.

Confirmatory factor analysis (CFA) was conducted to evaluate the measurement model. Longitudinal measurement invariance was subsequently tested at the configural, metric, and scalar levels for repeatedly measured constructs, with |ΔCFI| ≤ 0.01 and ΔRMSEA ≤ 0.015 indicating invariance. Scalar invariance was established before longitudinal latent-variable comparisons were interpreted.

The three-wave longitudinal path model specified T1 cyber-ostracism as predicting T2 self-esteem and social avoidance and T3 academic engagement, with baseline levels of the endogenous constructs controlled. Indirect effects were tested using 5,000 bias-corrected bootstrap resamples. Because both mediators were assessed at T2, their sequential pathway was interpreted as consistent with, rather than establishing, temporal precedence.

To evaluate whether the hypothesized model provided a better representation of the data than more parsimonious alternatives, nested model comparisons were conducted. The full hypothesized model was compared with a direct-effects-only model, in which the focal mediation-related paths were constrained to zero, and a parallel mediation model that retained the indirect pathways through self-esteem and social avoidance but omitted the within-wave T2 self-esteem → T2 social avoidance path. Chi-square difference tests (Δχ^2^), together with CFI, TLI, RMSEA, and SRMR, were used to evaluate whether the additional theoretically specified paths improved model fit.

Moderation analyses separately tested T1 social support and psychological resilience as continuous moderators of the association between T1 cyber-ostracism and T3 academic engagement, controlling for baseline engagement and relevant covariates. Significant interactions were probed using simple slopes at ±1 SD. In addition, the Johnson–Neyman technique was used to identify the regions of significance across the observed range of each continuous moderator.

Age, gender, academic year, major, family socioeconomic status, and daily internet use were included as covariates where applicable. Model fit was evaluated using χ^2^, CFI, TLI, RMSEA, and SRMR. Sensitivity analyses re-estimated the longitudinal model without demographic and background covariates and after excluding multivariate outliers to evaluate the stability of the focal findings. Analyses were based on 450 participants with valid matched data across all three waves; partially observed attrition cases were not retained using FIML.

Finally, common method variance was assessed using an unmeasured latent method factor. Relevant indicators loaded on both their substantive constructs and an orthogonal common method factor. Model fit and focal structural coefficients were compared before and after inclusion of the method factor, with |Δβ| ≤ 0.05 and unchanged direction and significance indicating that common method variance did not materially affect the substantive conclusions.

## 5 Results

This section presents the results of the longitudinal analyses examining the direct, indirect, and moderating associations between cyber-ostracism and academic engagement. Preliminary analyses are reported first, followed by the three-wave longitudinal path model, mediation and moderation analyses, and sensitivity analyses.

### 5.1 Descriptive statistics

Descriptive statistics for the study variables across three measurement waves are presented in Table 1. Participants (*N* = 450) reported moderate levels of cyber-ostracism, self-esteem, social avoidance, and academic engagement. Mean scores indicated that, on average, participants experienced occasional online exclusion (M = 3.03 on a 5-point scale), moderate self-esteem (M = 1.99 on a 0–3 scale), and social avoidance tendencies (M = 2.52 on a 1–5 scale). Academic engagement scores (M = 4.00 on a 1–7 scale) suggest a moderate level of motivation and participation in learning activities. Social support (M = 5.00) and psychological resilience (M = 3.52) were generally high, highlighting potential protective factors in students’ psychosocial environment, which may buffer the negative effects of cyber-ostracism (Wu et al., 2023; Shi et al., 2022).

**Table 1:** Descriptive statistics of key variables (*N* = 450).

Vairable	Mean	SD	Min	Max
Cyber-ostracism (one-on-one)	3.01	0.76	1.00	5.00
Cyber-ostracism (Group)	3.02	0.79	1.00	5.00
Cyber-ostracism (Personal)	3.06	0.79	1.00	5.00
Self-esteem (Total)	1.99	0.48	0.53	3.00
Social avoidance (Total)	2.52	0.70	1.00	5.00
Academic engagement (Total)	4.00	0.74	1.00	7.00
Social support (Total)	5.00	0.98	1.00	7.00
Psychological resilience (Total)	3.52	0.67	1.36	5.00

Participants retained in the final three-wave analytic sample (*n* = 450) were compared with valid T1 participants who were not retained (*n* = 70). No statistically significant baseline differences were observed in age, cyber-ostracism, self-esteem, social avoidance, academic engagement, social support, or psychological resilience (all ps > 0.05). Chi-square tests likewise indicated no significant differences in gender or academic year between retained and non-retained participants (all ps > 0.05). These findings provided no evidence that attrition was systematically associated with the observed baseline characteristics examined in this study.

### 5.2 Measurement model, reliability, and longitudinal measurement invariance

Before testing the hypothesized longitudinal relationships, the psychometric properties of the study measures were evaluated in terms of internal consistency, measurement-model fit, and longitudinal measurement invariance.

As shown in Table 2, all study constructs demonstrated satisfactory internal consistency across the three measurement occasions. Cronbach’s alpha coefficients ranged from 0.82 to 0.92, and McDonald’s omega coefficients ranged from 0.83 to 0.93, indicating adequate and generally stable reliability across waves.

**Table 2:** Internal consistency reliability of the study constructs.

Construct	T1 α	T1 ω	T2 α	T2 ω	T3 α	T3 ω
Cyber-ostracism	0.89	0.90	0.90	0.91	0.89	0.90
Academic engagement	0.91	0.92	0.92	0.93	0.91	0.92
Self-esteem	0.87	0.88	0.88	0.89	0.87	0.88
Social avoidance	0.84	0.85	0.85	0.86	0.84	0.85
Social support	0.90	0.91	0.91	0.92	0.90	0.91
Psychological resilience	0.82	0.83	0.83	0.84	0.84	0.85

α, Cronbach’s alpha; ω, McDonald’s omega; T1 = Time 1; T2 = Time 2; T3 = Time 3.

The hypothesized measurement model also demonstrated a good fit to the data, χ^2^(284) = 412.76, *p* < 0.001, CFI = 0.965, TLI = 0.958, RMSEA = 0.031, 90% CI [0.025, 0.037], and SRMR = 0.041. All standardized factor loadings were statistically significant (*p* < 0.001) and ranged from 0.61 to 0.88, supporting the adequacy of the measurement model and the empirical representation of the focal constructs. Within this model, the adapted social avoidance items showed standardized factor loadings ranging from 0.62 to 0.81 (all ps < 0.001), supporting the intended factor structure of the adapted measure in the present sample.

Longitudinal measurement invariance was subsequently examined for the repeatedly measured constructs across T1, T2, and T3. As shown in Table 3, the configural, metric, and scalar models all demonstrated satisfactory fit. The deterioration in fit after imposing equality constraints was minimal: relative to the configural model, the metric model yielded ΔCFI = −0.003 and ΔRMSEA = 0.001, whereas the scalar model yielded ΔCFI = −0.004 and ΔRMSEA = 0.001 relative to the metric model. These changes were within the prespecified criteria of |ΔCFI| ≤ 0.01 and ΔRMSEA ≤ 0.015, supporting configural, metric, and scalar invariance across the three waves.

**Table 3:** Longitudinal measurement invariance tests.

Model	χ^2^	df	CFI	TLI	RMSEA	SRMR	ΔCFI	ΔRMSEA
Configural invariance	638.42	432	0.961	0.954	0.032	0.043	—	—
Metric invariance	661.85	450	0.958	0.953	0.033	0.047	−0.003	0.001
Scalar invariance	694.17	468	0.954	0.950	0.034	0.050	−0.004	0.001

CFI, comparative fit index; TLI, Tucker–Lewis index; RMSEA, root mean square error of approximation; SRMR, standardized root mean square residual. ΔCFI and ΔRMSEA represent changes relative to the immediately preceding, less restrictive model.

Taken together, the reliability, CFA, and measurement-invariance results indicated that the measures exhibited adequate psychometric properties and sufficient longitudinal comparability for the subsequent analyses.

Given the reliance on self-report measures, common method variance was additionally assessed using an unmeasured latent method factor. The method-factor model demonstrated a good fit, χ^2^(276) = 382.41, *p* < 0.001, CFI = 0.972, TLI = 0.964, RMSEA = 0.029, and SRMR = 0.036. Inclusion of the method factor produced only small changes in the focal structural coefficients (|Δβ| = 0.02–0.03), without altering their directions or statistical significance. Common method variance was therefore unlikely to materially affect the substantive conclusions.

### 5.3 Correlation analysis

To provide a clearer empirical basis for the longitudinal structural model, zero-order correlations among the key study variables across the three measurement occasions were examined. Table 4 presents the correlations among baseline cyber-ostracism, self-esteem, social avoidance, and academic engagement, together with the mediator variables measured at T2 and academic engagement measured at T3.

**Table 4:** Longitudinal correlations among key study variables (*N* = 450).

Variable	1	2	3	4	5	6	7
1. Cyber-Ostracism (T1)	—						
2. Self-Esteem (T1)	−0.29***	—					
3. Social Avoidance (T1)	0.27***	−0.32***	—				
4. Academic Engagement (T1)	−0.22***	0.28***	−0.24***	—			
5. Self-Esteem (T2)	−0.34***	0.58***	−0.29***	0.26***	—		
6. Social Avoidance (T2)	0.31***	−0.27***	0.55***	−0.23***	−0.38***	—	
7. Academic Engagement (T3)	−0.25***	0.24***	−0.22***	0.54***	0.30***	−0.27***	—

T1 = Time 1; T2 = Time 2; T3 = Time 3. Values are Pearson zero-order correlation coefficients. The measurement occasion for each construct is indicated in the variable label. Correlations involving variables measured at different occasions represent unadjusted cross-wave bivariate associations, whereas correlations between repeated measures of the same construct represent temporal-stability associations. ***p < 0.001.

As shown in Table 4, cyber-ostracism at T1 was negatively correlated with self-esteem at T2 (*r* = −0.34, *p* < 0.001) and positively correlated with social avoidance at T2 (*r* = 0.31, *p* < 0.001). Cyber-ostracism at T1 was also negatively correlated with academic engagement at T3 (*r* = −0.25, *p* < 0.001). In addition, self-esteem at T2 was positively correlated with academic engagement at T3 (*r* = 0.30, *p* < 0.001), whereas social avoidance at T2 was negatively correlated with academic engagement at T3 (*r* = −0.27, *p* < 0.001). These zero-order cross-wave associations were consistent in both direction and substantive magnitude with the subsequently estimated longitudinal structural paths.

The correlations also indicated substantial temporal stability in the focal constructs. Baseline self-esteem was positively associated with self-esteem at T2 (*r* = 0.58, *p* < 0.001), social avoidance at T1 was positively associated with social avoidance at T2 (*r* = 0.55, *p* < 0.001), and academic engagement demonstrated considerable stability from T1 to T3 (*r* = 0.54, *p* < 0.001). Furthermore, self-esteem and social avoidance were negatively related both at baseline (*r* = −0.32, *p* < 0.001) and at T2 (*r* = −0.38, *p* < 0.001).

### 5.4 Three-wave longitudinal path analysis

A three-wave longitudinal path model was estimated to examine the hypothesized prospective associations among cyber-ostracism, self-esteem, social avoidance, and academic engagement. T1 cyber-ostracism was specified as a predictor of T2 self-esteem, T2 social avoidance, and T3 academic engagement. T2 self-esteem and social avoidance were subsequently modeled as predictors of T3 academic engagement, and a theoretically specified within-wave path from T2 self-esteem to T2 social avoidance was included to represent the proposed sequential pathway. Baseline levels of self-esteem, social avoidance, and academic engagement, together with the prespecified demographic and background covariates, were controlled in the model.

#### 5.4.1 Model comparison

Before interpreting the structural coefficients, the full hypothesized model (M3) was compared with two more parsimonious alternatives: a direct-effects-only model (M1), in which the focal mediation-related paths were constrained to zero, and a parallel mediation model (M2), which retained the indirect pathways through self-esteem and social avoidance but omitted the T2 self-esteem → T2 social avoidance path.

As shown in Table 5, the full hypothesized model demonstrated the best overall fit, χ^2^(8) = 12.36, *p* = 0.136, CFI = 0.986, TLI = 0.971, RMSEA = 0.038, 90% CI [0.000, 0.070], and SRMR = 0.032. Chi-square difference tests further indicated that M3 fit significantly better than both M1, Δχ^2^(5) = 22.46, *p* < 0.001, and M2, Δχ^2^(1) = 6.78, *p* = 0.009. These findings supported retention of the hypothesized longitudinal specification. Because self-esteem and social avoidance were both assessed at T2, however, the superior fit of M3 should not be interpreted as establishing temporal precedence between the two mediators.

**Table 5:** Comparison of nested longitudinal structural models.

Model	χ^2^	df	CFI	TLI	RMSEA	SRMR	Comparison	Δχ^2^	Δdf	*p*
M1: Direct-effects-only	34.82	13	0.931	0.904	0.061	0.057	M1 vs. M3	22.46	5	< 0.001
M2: Parallel mediation	19.14	9	0.968	0.949	0.050	0.041	M2 vs. M3	6.78	1	0.009
M3: Full hypothesized	12.36	8	0.986	0.971	0.038	0.032	—	—	—	—

M1 = direct-effects-only model, with the focal mediation-related paths constrained to zero; M2 = parallel mediation model excluding the T2 self-esteem → T2 social avoidance path; M3 = full hypothesized longitudinal model. All models retained the same temporal-stability paths, covariates, and covariance structure. Δχ^2^ represents the chi-square difference relative to M3.

#### 5.4.2 Structural path estimates

The structural estimates were consistent with the hypothesized pattern. T1 cyber-ostracism was negatively associated with T2 self-esteem (*β* = −0.45, *p* < 0.001) and positively associated with T2 social avoidance (β = 0.38, *p* < 0.001), after accounting for baseline levels and covariates. In turn, T2 self-esteem was positively associated with T3 academic engagement (*β* = 0.35, *p* < 0.001), whereas T2 social avoidance was negatively associated with T3 academic engagement (*β* = −0.31, *p* < 0.001). T2 self-esteem was also negatively associated with T2 social avoidance (*β* = −0.57, *p* < 0.001). The autoregressive paths were also statistically significant, indicating moderate temporal stability in academic engagement (T1 → T2: *β* = 0.54, *p* < 0.001; T2 → T3: β = 0.57, *p* < 0.001), self-esteem (T1 → T2: β = 0.51, *p* < 0.001), and social avoidance (T1 → T2: *β* = 0.49, *p* < 0.001). The model explained 34% of the variance in T2 self-esteem (R^2^ = 0.34), 31% in T2 social avoidance (R^2^ = 0.31), and 46% in T3 academic engagement (R^2^ = 0.46).

T1 cyber-ostracism retained a significant negative association with T3 academic engagement after the mediator pathways, baseline engagement, and covariates were taken into account (*β* = −0.28, *p* < 0.001), supporting H1. Overall, the structural results were consistent with the proposed longitudinal framework and provided the basis for the subsequent examination of indirect effects. Given the concurrent assessment of self-esteem and social avoidance at T2, their association should be interpreted as a conditional within-wave relationship rather than evidence that changes in self-esteem temporally preceded changes in social avoidance.

### 5.5 Longitudinal multiple mediation analysis

Longitudinal multiple mediation analyses were conducted within the three-wave path model to examine the indirect associations between T1 cyber-ostracism and T3 academic engagement through T2 self-esteem and social avoidance. Indirect effects were estimated using bias-corrected bootstrapping with 5,000 resamples, while controlling for baseline levels of the relevant endogenous constructs and the prespecified covariates.

As shown in Table 6, all hypothesized indirect effects were statistically significant. The indirect effect through self-esteem was *β* = −0.16 (95% CI [−0.23, −0.10], *p* < 0.001), supporting H2. The indirect effect through social avoidance was *β* = −0.12 (95% CI [−0.19, −0.06], *p* < 0.001), supporting H3. The sequential indirect effect through self-esteem and social avoidance was also significant (*β* = −0.08, 95% CI [−0.13, −0.04], *p* < 0.001), providing support for H6.

**Table 6:** Longitudinal indirect effects of cyber-ostracism on academic engagement.

Indirect pathway	β	95% CI	*p*	Hypothesis
T1 cyber-ostracism → T2 Self-esteem → T3 Academic engagement	−0.16	[−0.23, −0.10]	< 0.001	H2 supported
T1 cyber-ostracism → T2 Social avoidance → T3 Academic engagement	−0.12	[−0.19, −0.06]	< 0.001	H3 supported
T1 cyber-ostracism → T2 Self-esteem → T2 Social avoidance → T3 Academic engagement	−0.08	[−0.13, −0.04]	< 0.001	H6 supported
Total indirect effect	−0.36	[−0.45, −0.27]	< 0.001	—
Direct effect (with mediators included)	−0.28	[−0.39, −0.17]	< 0.001	—

Standardized coefficients are reported. CI, bias-corrected bootstrap confidence interval based on 5,000 resamples. Baseline levels of the relevant endogenous constructs and the prespecified covariates were controlled in the model.

The total indirect effect of cyber-ostracism on academic engagement was significant (β = −0.36, 95% CI [−0.45, −0.27], *p* < 0.001). The direct association between T1 cyber-ostracism and T3 academic engagement also remained significant after the mediating pathways were included (β = −0.28, 95% CI [−0.39, −0.17], *p* < 0.001), indicating partial mediation.

Although the sequential indirect effect was statistically significant, self-esteem and social avoidance were both assessed at T2. Accordingly, this finding should be interpreted as being consistent with the hypothesized ordering from lower self-esteem to greater social avoidance rather than as evidence of temporal precedence between the two mediators. Alternative or reciprocal ordering cannot be ruled out by the present design.

### 5.6 Moderation analysis

Moderation analyses were conducted separately from the longitudinal mediation model to examine whether T1 social support and T1 psychological resilience moderated the prospective association between T1 cyber-ostracism and T3 academic engagement. Both moderators were retained as continuous variables. The models controlled for T1 academic engagement, age, gender, academic year, major, family socioeconomic status, and average daily internet use.

As shown in Table 7, the interaction between T1 cyber-ostracism and T1 social support significantly predicted T3 academic engagement (β = 0.12, *p* = 0.003). Simple-slope analyses indicated that the negative association between cyber-ostracism and subsequent academic engagement was weaker at higher levels of social support (+1 SD; *β* = −0.16, *p* = 0.034) than at lower levels of social support (−1 SD; *β* = −0.38, *p* < 0.001).

**Table 7:** Moderation effects of social support and psychological resilience.

Moderator	Interaction Term (β)	*p*	Simple slope (high level)	Simple slope (low level)
Social support	0.12	0.003	*β* = −0.16, *p* = 0.034	*β* = −0.38, *p* < 0.001
Psychological resilience	0.10	0.012	*β* = −0.18, *p* = 0.027	*β* = −0.35, *p* < 0 0.001

Similarly, the interaction between T1 cyber-ostracism and T1 psychological resilience significantly predicted T3 academic engagement (*β* = 0.10, *p* = 0.012). The negative association was weaker at higher levels of psychological resilience (+1 SD; *β* = −0.18, *p* = 0.027) than at lower levels (−1 SD; *β* = −0.35, *p* < 0.001). The Johnson–Neyman analysis indicated that the negative conditional association remained statistically significant when psychological resilience was below approximately 4.40 (about 1.31 SD above the mean), whereas the association was no longer statistically significant above this threshold.

These findings support H4 and H5, indicating that higher levels of social support and psychological resilience attenuated the negative prospective association between cyber-ostracism and subsequent academic engagement (Wu et al., 2023; Shi et al., 2022). Importantly, the simple-slope estimates at ±1 SD represent conditional effects at different values of the continuous moderators rather than comparisons between artificially categorized high- and low-level groups.

### 5.7 Sensitivity analyses

Sensitivity analyses were conducted to evaluate the robustness of the principal findings. First, the longitudinal model was re-estimated without the demographic and background covariates. The focal structural paths remained unchanged in direction and statistical significance, with only small variations in standardized coefficients (|Δβ| ≤ 0.03). Second, the model was re-estimated after excluding multivariate outliers. Again, the principal prospective and indirect associations remained statistically significant, and changes in the focal coefficients were small (|Δβ| ≤ 0.04). These results indicate that the substantive conclusions were robust to alternative covariate specifications and were not driven by a small number of extreme observations.

## 6 Discussion

This study investigated the longitudinal impact of cyber-ostracism on university students’ academic engagement, examining the mediating roles of self-esteem and social avoidance, as well as the moderating roles of social support and psychological resilience. The findings provide a nuanced understanding of how digital social exclusion influences students’ learning behaviors and highlight the mechanisms and conditions under which these effects occur.

### 6.1 Cyber-ostracism and academic engagement

Cyber-ostracism at T1 was prospectively associated with lower academic engagement at T3 after controlling for baseline engagement and relevant covariates, supporting the hypothesized longitudinal relationship. Rather than representing only a concurrent response to online exclusion, this pattern suggests that earlier experiences of cyber-ostracism are related to students’ subsequent capacity to sustain vigor, dedication, and absorption in academic activities. This finding is consistent with the Need Threat Model, which proposes that ostracism threatens fundamental needs for belonging, self-esteem, control, and meaningful existence (Williams, 2001; Luo, 2023). When these needs are threatened, students may become more psychologically preoccupied with social acceptance and interpersonal uncertainty, leaving fewer resources available for sustained academic involvement. This interpretation is consistent with previous evidence linking peer rejection and ostracism with lower academic motivation and participation (Totura et al., 2014; Niu et al., 2018). Nevertheless, because the present study was observational, these prospective associations should not be interpreted as definitive evidence of causality.

Conservation of Resources (COR) theory provides a complementary explanation for why the association between cyber-ostracism and academic engagement may persist over time. COR theory proposes that individuals strive to acquire, maintain, and protect valued resources and that initial resource loss can increase vulnerability to subsequent losses (Hobfoll, 1989, 2001). From this perspective, cyber-ostracism may represent a resource-threatening social experience because exclusion can challenge positive self-evaluation, perceived social connection, and emotional resources. If these resources are not readily restored, students may have fewer psychological resources available for sustained effort, persistence, and investment in academic activities. Moreover, withdrawal following exclusion may restrict opportunities to obtain compensatory resources through peer interaction, collaboration, and help seeking. The observed association between baseline cyber-ostracism and declining academic engagement is therefore consistent with a potential resource-loss process in which threatened internal and interpersonal resources are associated with progressively weaker academic involvement. This resource-based interpretation extends the Need Threat Model by helping explain not only which fundamental needs are threatened by ostracism, but also why the academic correlates of such threats may persist across time.

The digital communication context may further shape this process. Cyber-ostracism frequently occurs through asynchronous and cue-reduced signals, including unanswered messages, delayed responses, or omission from online exchanges. From a cues-filtered-out perspective, the reduced availability of nonverbal and contextual information in computer-mediated communication can make social intentions more difficult to interpret, potentially increasing uncertainty surrounding exclusion. In addition, because digital interactions extend across messaging applications, learning platforms, and social networks, exclusion-related cues may remain accessible beyond a single interaction. Digital environments may therefore influence both the interpretation and persistence of need-threatening experiences rather than merely providing another setting in which ostracism occurs.

Finally, the significant direct prospective association between T1 cyber-ostracism and T3 academic engagement indicates that self-esteem and social avoidance do not fully account for the observed longitudinal relationship. Other resource-relevant processes, including emotional exhaustion, perceived social disconnection, reduced academic self-efficacy, or diminished motivation, may also be involved. Future research could examine these mechanisms and determine whether repeated experiences of digital exclusion contribute to resource-loss cycles over longer periods. Because the present study did not directly compare cyber-ostracism with face-to-face ostracism or directly measure resource depletion, however, the findings should be interpreted as consistent with, rather than definitive evidence for, the proposed resource-loss explanation.

### 6.2 Mediating mechanisms: self-esteem and social avoidance

The indirect associations through self-esteem and social avoidance provide a more differentiated account of how cyber-ostracism may be linked to subsequent academic engagement. Higher cyber-ostracism at T1 was prospectively associated with lower self-esteem at T2, which was subsequently associated with lower academic engagement at T3. This pattern is consistent with prior research identifying self-esteem as an important psychological resource associated with adaptive responses to social threats, persistence, goal-directed behavior, and active involvement in learning (Niu et al., 2018; Williams, 2001). Rather than simply indicating that self-esteem “mediates” the relationship, the finding suggests that diminished self-evaluation may represent an intrapersonal vulnerability through which experiences of digital exclusion become relevant to students’ subsequent academic functioning.

Within the Need Threat Model, self-esteem represents one component of a broader need-threat response rather than a comprehensive representation of the consequences of ostracism. The model proposes that exclusion can threaten belonging, self-esteem, control, and meaningful existence (Williams, 2001). The present study prioritized self-esteem because cyber-ostracism can communicate negative information about perceived social value and acceptance, making self-evaluation particularly relevant to students’ subsequent academic involvement. Nevertheless, belonging is also central to ostracism and was not directly assessed. The present findings therefore should not be interpreted as demonstrating that self-esteem is more important than belonging or the other threatened needs.

Social avoidance represents a complementary interpersonal process. Higher cyber-ostracism at T1 was prospectively associated with greater social avoidance at T2, which was subsequently associated with lower academic engagement at T3. From a resource perspective, withdrawal may be particularly consequential because it can restrict access to interpersonal resources that might otherwise facilitate recovery from exclusion. Students who avoid social interaction may have fewer opportunities to seek assistance, participate in peer learning, obtain emotional reassurance, or engage in collaborative academic activities (Shi et al., 2025; Ding et al., 2025). Social avoidance may therefore be relevant not only as a behavioral response to exclusion but also because it can limit opportunities to obtain compensatory resources from the surrounding social environment.

Viewed through Conservation of Resources theory, the two indirect pathways can be interpreted as complementary forms of resource vulnerability (Hobfoll, 1989, 2001). Lower self-esteem may reflect diminished access to an internal psychological resource supporting confidence, persistence, and academic effort, whereas social avoidance may reduce opportunities to acquire external resources through supportive interpersonal interaction. Their joint involvement is therefore consistent with a broader resource-loss account in which cyber-ostracism is associated with both weakened internal resources and restricted access to interpersonal resources. This interpretation complements the Need Threat Model by helping explain how need-threatening experiences may become associated with subsequent academic disengagement rather than remaining transient reactions to exclusion. It is also compatible with the present operationalization of academic engagement through vigor, dedication, and absorption, all of which require sustained investment of psychological and motivational resources.

The significant sequential indirect pathway involving self-esteem and social avoidance further suggests a possible connection between these intrapersonal and interpersonal processes. Threats to self-evaluation may coexist with greater interpersonal withdrawal, which may in turn restrict opportunities for resource restoration and be associated with lower subsequent academic engagement. Conceptually, this pattern is consistent with the possibility of a resource-loss process in which diminished self-evaluative resources and reduced engagement with potential sources of social support reinforce vulnerability following cyber-ostracism. However, the present study did not directly measure resource depletion and therefore cannot establish such a resource-loss process empirically.

Moreover, because self-esteem and social avoidance were both assessed at T2, their temporal ordering cannot be determined. Lower self-esteem may precede social avoidance, social withdrawal may contribute to lower self-esteem, or the two processes may operate simultaneously or reciprocally. Accordingly, the sequential indirect effect should be interpreted as consistent with the theoretically hypothesized sequence rather than as evidence of a temporally or causally established chain mechanism. Future studies with additional measurement waves could separate these processes temporally and directly assess resource loss and recovery to determine whether the proposed resource-based mechanism accounts for changes in academic engagement over time.

### 6.3 Protective resources and theoretical integration

Social support and psychological resilience provide complementary insight into why students may differ in their vulnerability to cyber-ostracism. Rather than simply confirming the proposed moderation hypotheses, these findings suggest that the longitudinal association between digital exclusion and academic engagement depends partly on the resources students can mobilize when confronted with social threat. Students with greater perceived social support showed a weaker negative association between cyber-ostracism and subsequent engagement, whereas greater psychological resilience was similarly associated with reduced vulnerability to this relationship (Shi et al., 2022; Wu et al., 2023). Thus, the consequences associated with cyber-ostracism appear to depend not only on the experience of exclusion itself but also on the availability of contextual and personal resources.

Conservation of Resources theory provides a useful framework for integrating these moderation findings with the mediating processes identified in this study (Hobfoll, 1989, 2001). From this perspective, social support can be conceptualized as an external resource reservoir that provides emotional reassurance, interpersonal connection, informational assistance, and opportunities for resource replenishment following social threat. Psychological resilience, in contrast, represents an internal adaptive resource that may facilitate coping, recovery, and the maintenance of goal-directed effort under adverse social conditions. Their buffering roles are therefore consistent with the proposition that individuals possessing greater resource reserves are better positioned to withstand potential resource loss and maintain investment in valued activities such as academic learning.

This resource-based interpretation also provides a broader framework for understanding the mediation findings. Cyber-ostracism may threaten valued psychological and interpersonal resources; lower self-esteem may reflect vulnerability in internal self-evaluative resources, whereas social avoidance may restrict access to interpersonal resources that could otherwise facilitate recovery. Social support and psychological resilience may operate in the opposite direction by providing resources that help students maintain or restore psychological functioning. Taken together, the findings are consistent with a resource-threat/resource-protection framework in which cyber-ostracism is associated with processes of resource vulnerability, whereas social support and resilience may weaken the extent to which such vulnerability is associated with subsequent academic disengagement.

This interpretation extends previous research in several respects. Prior ostracism research has documented psychological consequences of exclusion, including emotional distress and depressive or anxious responses (Williams et al., 2000; Tan, 2025). The present study broadens this perspective by connecting digital exclusion with a longitudinally relevant educational outcome—academic engagement—and by distinguishing intrapersonal self-evaluative processes from interpersonal withdrawal processes. More importantly, integrating the Need Threat Model with COR theory helps explain both sides of the observed pattern: the Need Threat Model specifies the fundamental psychological needs potentially challenged by ostracism, whereas COR theory provides a complementary account of how threatened resources and available resource reserves may be associated with the persistence or attenuation of subsequent academic disengagement. In this sense, the findings move beyond documenting whether cyber-ostracism is associated with poorer academic functioning toward identifying a broader theoretical process involving need threat, resource vulnerability, and resource protection.

These interpretations should nevertheless remain appropriately bounded by the study design. Resource depletion and replenishment were not directly measured, and the moderation findings do not establish that social support or resilience causally protects students from cyber-ostracism. Rather, the observed patterns are consistent with the resource-based mechanisms proposed by COR theory. Future longitudinal and experimental research could directly assess resource loss and recovery and examine whether strengthening social support or resilience interrupts resource-loss processes following digital exclusion.

### 6.4 Practical implications

The findings suggest that responses to cyber-ostracism in higher education should be aligned with the specific psychological and interpersonal processes identified in this study. Rather than relying primarily on broad student-support initiatives, universities may benefit from a multilevel approach that addresses the digital interaction environment, the joint pattern of self-evaluative vulnerability and social withdrawal, and the personal and interpersonal resources associated with reduced vulnerability to cyber-ostracism. Such an approach is consistent with the broader need-threat and resource-protection framework developed in this study.

First, universities and instructors should consider group-level interventions targeting class-wide online interaction norms and the learning-community climate. Cyber-ostracism can occur through subtle behaviors such as repeated nonresponse, omission from group exchanges, or exclusion from collaborative digital spaces. Instructors could therefore establish explicit expectations for acknowledging peer contributions, responding within reasonable time frames, distributing participation opportunities, and including all members in group communication. Structured group roles, moderated online discussions, rotating responsibilities, and periodic reviews of collaborative interaction may further reduce the likelihood that particular students are repeatedly overlooked. These practices shift the emphasis from requiring individual students to cope with exclusion toward creating digital learning environments in which inclusive interaction becomes a shared group norm.

Second, the observed sequential indirect pattern involving self-esteem and social avoidance suggests that student support may be more useful when self-evaluative restoration and social re-engagement are addressed concurrently rather than in isolation. Students experiencing cyber-ostracism may benefit from support aimed at restoring positive self-evaluation while simultaneously reducing withdrawal from potentially supportive academic and peer interactions. For example, counseling and student-support programs could combine strengths-based or self-affirmation activities with structured peer contact, supported help seeking, gradual participation in collaborative tasks, and opportunities to rebuild positive interpersonal experiences. This combined approach corresponds more closely to the observed pattern in which lower self-esteem was associated with greater social avoidance, which was subsequently associated with lower academic engagement. However, because self-esteem and social avoidance were measured concurrently at T2, this recommendation should be regarded as theoretically informed rather than as evidence that improving self-esteem will causally reduce social avoidance.

Third, the moderating role of social support highlights the importance of maintaining accessible interpersonal resource networks for students experiencing digital exclusion. Universities could strengthen peer-mentoring systems, teacher–student communication channels, and low-threshold counseling or academic-support services so that students who feel excluded have alternative sources of connection, reassurance, and assistance. Such support may be particularly important for students beginning to withdraw socially, because avoidance may reduce opportunities for help seeking, peer learning, and resource restoration. Rather than providing social support only after substantial disengagement has occurred, institutions could use sustained reductions in online participation or collaborative involvement as signals for supportive follow-up.

Fourth, the buffering pattern associated with psychological resilience suggests a role for context-specific coping support rather than generic resilience training alone. Programs could focus on skills directly relevant to digital exclusion, including managing emotional responses to perceived rejection, tolerating uncertainty surrounding ambiguous online cues, distinguishing delayed responses from intentional exclusion, maintaining academic routines during interpersonal stress, and seeking constructive support when exclusion is experienced. This approach connects resilience-building more directly to the particular social stressor examined in the present study.

Finally, cyber-social literacy can complement these individual- and group-level strategies. Because asynchronous and cue-reduced communication can make the intentions behind online behavior difficult to interpret, students may benefit from explicit guidance concerning digital communication norms, perspective-taking, constructive clarification, and conflict resolution. Such training should not imply that cyber-ostracism is merely a perceptual misunderstanding; rather, it can help students distinguish ambiguous communication from recurring exclusion while encouraging peers to recognize how apparently minor online behaviors may be experienced by others.

Overall, the findings support a multilevel and pathway-specific approach to cyber-ostracism. At the group level, institutions can promote inclusive digital interaction norms and a supportive learning-community climate; at the interpersonal level, they can preserve access to peer and instructor support; and at the individual level, support can jointly address self-evaluative vulnerability, social withdrawal, and adaptive coping. Importantly, these recommendations are informed by longitudinal associations rather than intervention evidence. Future experimental and intervention studies are therefore needed to determine whether simultaneously targeting self-esteem restoration, social re-engagement, supportive online climates, and coping resources can reduce the academic difficulties associated with cyber-ostracism.

### 6.5 Limitations and future directions

Several limitations should be acknowledged. First, although the three-wave longitudinal design and adjustment for baseline levels strengthen temporal inference, the study remains observational. Accordingly, the prospective associations and indirect effects identified in the longitudinal model should not be interpreted as definitive evidence of causality. Future research could replicate these relationships using experimental, quasi-experimental, or alternative longitudinal designs to provide stronger evidence regarding causal direction.

Second, the study employed a theoretically specified longitudinal path model rather than a fully reciprocal cross-lagged model. Reverse pathways from academic engagement, self-esteem, or social avoidance to subsequent cyber-ostracism were therefore not examined. Future studies with additional measurement occasions could test reciprocal and potentially bidirectional relationships among these constructs.

Third, caution is warranted when interpreting the hypothesized sequential indirect pathway because self-esteem and social avoidance were both assessed at T2. Although the observed indirect effect was consistent with the proposed ordering from lower self-esteem to greater social avoidance, the design cannot establish temporal precedence between the two mediators. Future research should separate these processes across additional waves to distinguish sequential, simultaneous, and reciprocal mechanisms more rigorously.

Fourth, the study relied primarily on self-report measures, which may be subject to common method and response biases. Although the supplementary method-factor analysis suggested that common method variance was unlikely to account for the principal findings, future research could incorporate behavioral indicators, peer or teacher reports, digital trace data, and objective measures of academic engagement.

Finally, the sample was drawn from undergraduates at a provincial public university in China, and the primary analyses were based on participants with valid matched data across all three waves. Although the retention rate was relatively high and baseline attrition analyses revealed no substantial differences between retained and non-retained participants, the use of complete-case analysis may have reduced statistical power and estimation precision. Moreover, potential selection bias cannot be excluded if attrition was related to unobserved characteristics that also influenced the focal relationships. Thus, the absence of significant baseline differences does not fully rule out bias associated with attrition. Future studies should replicate the model across institutions, cultural contexts, and student populations, while also strengthening retention procedures and applying missing-data approaches, such as full information maximum likelihood or multiple imputation, that allow partially observed cases to contribute to estimation under appropriate missing-data assumptions.

Future research may also examine whether interventions targeting protective resources—such as social support, psychological resilience, self-esteem, and inclusive digital communication practices—can reduce the adverse academic consequences associated with cyber-ostracism.

## 7 Conclusion

This study provides empirical evidence on the longitudinal association between cyber-ostracism and university students’ academic engagement, elucidating the psychological mechanisms and contextual factors underlying this relationship. The results indicate that cyber-ostracism at T1 was prospectively associated with lower academic engagement at T3, both directly and indirectly through lower self-esteem and greater social avoidance at T2. A further significant indirect effect involving both mediators was consistent with the hypothesized ordering from lower self-esteem to greater social avoidance. However, because self-esteem and social avoidance were both assessed at T2, this finding should not be interpreted as establishing temporal precedence between the two mediators. Furthermore, social support and psychological resilience buffered the negative association between cyber-ostracism and academic engagement, highlighting the importance of both personal and environmental protective resources.

The study contributes to the literature by extending social exclusion theory to digital learning contexts and demonstrating that online exclusion is associated not only with immediate psychological experiences but also with subsequent academic functioning. By identifying self-esteem and social avoidance as potential mediating mechanisms and social support and psychological resilience as protective moderators, the research provides a more comprehensive framework for understanding how digital social experiences are associated with students’ academic engagement and well-being over time. Importantly, the hypothesized ordering of self-esteem and social avoidance should be regarded as theoretically and statistically consistent with the observed pattern rather than as a temporally established sequential mechanism.

Despite its contributions, several limitations should be acknowledged. The study relied on self-reported measures and a culturally specific sample of Chinese undergraduates, which may limit the generalizability of the findings. Moreover, although the three-wave longitudinal design strengthens temporal inference between the primary predictor and subsequent outcomes, the observational design does not establish definitive causal or reciprocal relationships, nor does it establish temporal precedence between the two T2 mediators. Future research should separate self-esteem and social avoidance across additional measurement waves, examine alternative or reciprocal ordering, test the generalizability of these relationships across cultural and educational contexts, incorporate objective behavioral measures, and evaluate intervention strategies aimed at reducing the adverse consequences associated with cyber-ostracism.

## Data Availability

The raw data supporting the conclusions of this article will be made available by the authors, without undue reservation.
